# Case report: Xeroderma pigmentosum Group A with erythropoietic protoporphyria in a young Chinese patient

**DOI:** 10.3389/fendo.2024.1418254

**Published:** 2024-07-26

**Authors:** Shu-hui Wu, Ting Xiao, Dan Zhao, Ying-hong Zeng, Ming-fang Zhu

**Affiliations:** ^1^ Department of Dermatology, The Second Affiliated Hospital of Hunan University of Chinese Medicine, Hunan, Changsha, China; ^2^ Department of Dermatology, Hunan Children’s Hospital, Hunan, Changsha, China; ^3^ Hunan Provincial Key Laboratory of Vascular Biology and Translational Medicine, Changsha, Hunan, China

**Keywords:** xeroderma pigmentosum, Group A, erythropoietic protoporphyria, protoporphyrin, case

## Abstract

Xeroderma pigmentosum is a rare autosomal recessive genodermatoses characterized by a deficiency in nucleotide excision repair. Erythropoietic protoporphyria is a rare inherited metabolic disease caused by the perturbation of heme. Xeroderma pigmentosum-erythropoietic protoporphyria is exceedingly rare. Hereby, we firstly report a young Chinese patient of xeroderma pigmentosum Group A with erythropoietic protoporphyria carrying an XPA Met214AsnfsTer7 frameshift mutation and a homozygous splicing mutation, c.315–48T>C, in the proband’s intron3 of FECH.

## Introduction

Xeroderma pigmentosum (XP) is a rare autosomal recessive genodermatoses, affecting an estimated 1/250,000–1/1000,000 of the population of Europe and of China (1). XP is characterized by severe photosensitivity, ophthalmological and neurological manifestations, and a significantly increased risk of cutaneous malignancies due to a deficiency in nucleotide excision repair caused by single-nucleotide mutations (2, 3). Reportedly, eight genes and their proteins (XPA, XPB, XPC, XPD, XPE, XPF, XPG, and XPV) on nucleotide excision repair are verified to play an indispensable role in the process of XP. Based on the specific mutations, XP can be further subdivided into eight genotypes, involving seven complementation groups (XP-A, XP-B, XP-C, XP-D, XP-E, XP-F, XP-G) and an XP-variant (XP-V) (4). The XP-C group is the most common subtype in the United States, Europe, and Africa whilst the XP-A group is the most common subtype in China and Japan (5). The different types of genotypes of XP patients have different clinical manifestations, manifesting in the degree of sensitivity to ultraviolet light. In addition to severe sunburn with blistering or persistent erythema on minimal sun exposure, XP-A patients develop progressive neurodegeneration, such as progressive intellectual impairment, isolated hyporeflexia, and so on (6). The highest degree of photosensitivity is the hallmark of the XP-A patients. Most individuals with XP-A have a manifestation of hyper- or hypopigmentation accompanied by dryness or atrophy in the ultraviolet-exposed areas.

Erythropoietic protoporphyria (EPP), a rare inherited metabolic disease caused by heme perturbation, is characterized by the accumulation of metal-free protoporphyrin IX (PPIX) in erythroid cells due to a partial deficiency in ferrochelatase (FECH). This leads to phototoxic reactions responsible for long-lasting painful photodermatosis after sun exposure (7). EPP is considered a photosensitive disease, presenting intense burning, tingling, and itching in sun-exposed areas. These symptoms may be followed by redness, swelling, or blanching, lasting from minutes to days (8). Interestingly, EPP and XP are the inherited cutaneous diseases, which both manifest the symptoms of photophobia. Here, we present a xeroderma pigmentosum group A patient with EPP who was once considered to be “protoporphyria?”. The whole-exome sequencing (WES) revealed a novel mutation in XPA (c.640dup/p.Met214AsnfsTer7), along with the pathogenic variants in FECH (c.315–48T > C/splicing).

## Narrative

This case involved a 12-year-old female Chinese child (born in 2011), whose parents were cousins. Notably, she has exhibited erythema and blister formation on UV-exposed areas, particularly the neck, face, and upper limbs since early childhood. Additionally, her parents observed the development of hyperpigmented and hypopigmented spots predominantly on sun-exposed regions following prolonged sun exposure. At the age of five, she sought clinical evaluation at the Second Xiangya Hospital of Central South University where she received a provisional diagnosis of ‘photosensitive dermatitis.’ Could this be indicative of porphyria? The histopathological examination of the skin biopsy specimen revealed abundant neutrophils and erythrocytes within the epidermis along with significant vacuolar degeneration accompanied by mild pigmentary incontinence as well as dilated and hyperemic dermal blood vessels.

**Figure 1:**
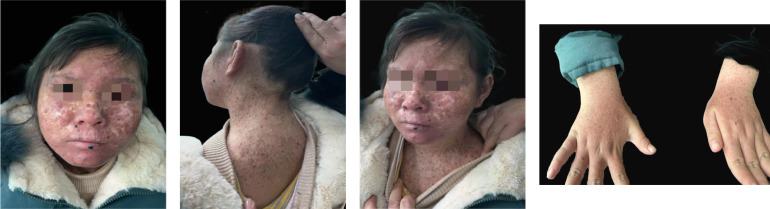
Clinical imaging.

**Figure 2:**
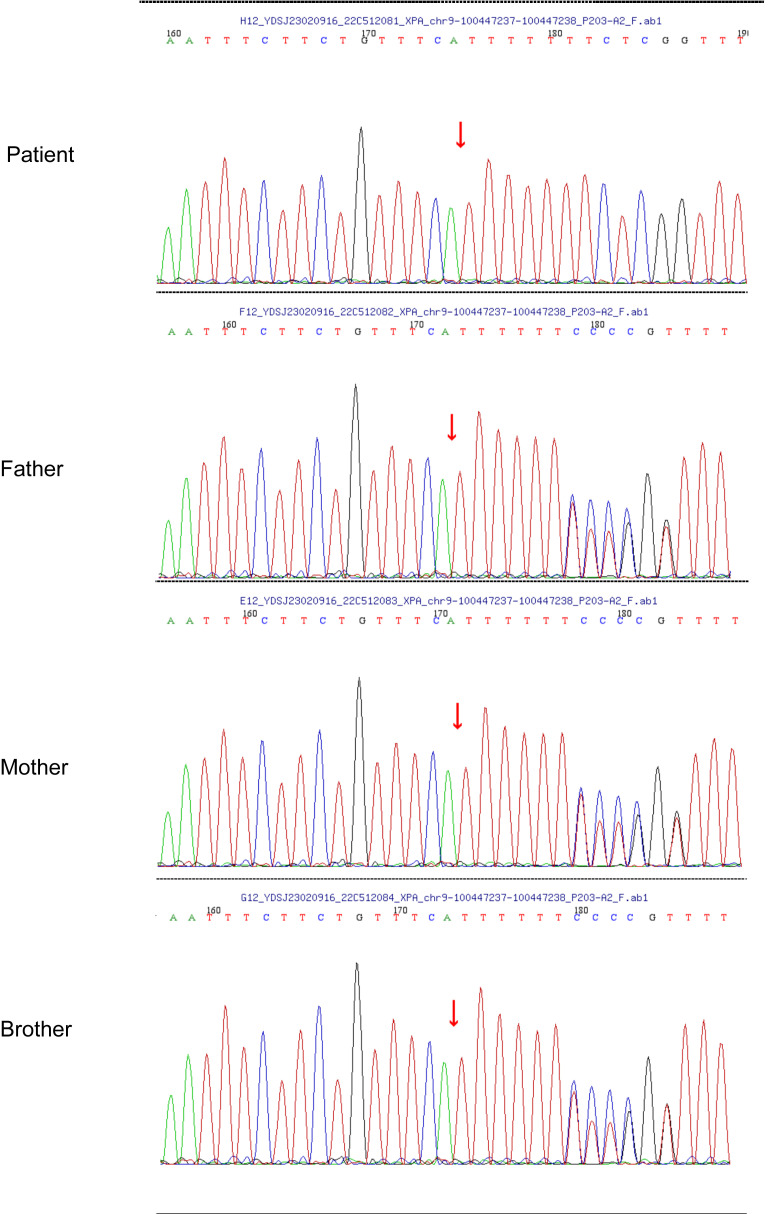
Sanger chromatogram demonstrating relevant mutations in XPA.

Approximately 1 year ago, she consulted the dermatology department of Hunan Children’s Hospital for a blackish nodule on the medial side of her left lower lip (**Figure 1**). Currently, the patient presents with prominent skin lesions and exhibits ocular abnormalities including photophobia, tearing, occasional redness, and discharge. Ophthalmic examination revealed irregular eyelids, corneal congestion, conjunctival congestion, and exotropia. In addition to ophthalmological features, neurological symptoms were observed in this case including cerebellar ataxia, cognitive impairment, and hearing impairment. Magnetic resonance imaging indicated delayed or dysplastic development of the cerebral hemisphere and cerebellum. Furthermore, numerous densely distributed brown pigmented patches and light pigmentary loss patches were observed on the face, neck, chest, back, and upper limbs. Dermatologically, a black nodule measuring approximately 0.3cm × 0.2cm under the right nostril, 0.5 cm × 0.5 cm on the lower lip, and another one measuring about 0.3 cm × 0.2 cm on left of the patient’s face (**Figure 1**) were visible respectively. The dermoscopy results suggested a possibility of malignancy; however, the patient declined further pathological examination. To confirm the diagnosis, genetic testing was performed after genetic counseling at the Center for Clinical Genomics in our hospital. Genomic DNA was extracted from the patient’s peripheral blood using a rapid extraction method and WES was conducted. The WES demonstrated a novel homozygous mutation of c.640dup, in the proband’s exon 5 of XPA (NM_000380.4), which induced the frameshift mutation (**Figure 2**). Further Sanger sequencing was performed on the patient’s parents and brother. The Sanger sequencing analysis confirmed that the detected mutation is present in heterozygous state in parents and brother. Besides the mutation of XPA, the homozygous splicing mutation, c.315–48T>C, in the proband’s intron3 of FECH(NM_000140.5) was observed.

## Discussion

XP is an autosomal recessive disease resulting from the mutation of nucleotide excision repair-related genes. Regrettably, there are currently no efficacious pharmacological interventions available to impede the progression of XP. The management strategies, such as sun avoidance, have a profound impact on individuals with XP.

In this reported case, it was confirmed that the disease-causing mutation was detected in XPA, which was verified to be the most common subtype in China (9). The detected pathogenic variant of the case resulted in the dysfunction of XPA. Reportedly, XPA plays a vital role in nucleotide excision repair (NER). The NER pathway is the most versatile repair pathway and is responsible for removing a wide variety of DNA-distorting lesions from the genome, including those induced by UV photolesions, chemical carcinogens, chemotherapeutic agents, or environmental genotoxins (10, 11). NER can be activated through one of two modes: the global-genome (GG-NER) pathway or the transcription-coupled (TC-NER) pathway. However, XPA, as a central scaffold protein that coordinates the assembly of repair complexes, is demonstrated to be associated with the recruitment of the 10-subunit transcription factor IIH (TFIIH) complex (12). In this case, the frameshift mutation at the 214rd methionine which affected the folding of the central domain of XPA, including the zinc finger, DNA- and RPA70-binding domains, caused the destabilization and inactivation of XPA. The inactivation of XPA disrupted the repair activity on the DNA-distorting lesions, which resulted in erythema, blisters, and lentiginous pigmentation in the exposed areas, as well as stunted physical and cognitive development. An epidemiological study demonstrated that XP is highly associated with the consanguinity level, which could be the main drive for XP (13). In this case, the parents with consanguineous relation are heterozygous and asymptomatic, which contributes to the higher chances of homozygous mutations in the next generation.

In addition, a c.315–48T>C mutation of FECH, the pathogenic variant of EPP, was observed in the case (14). Reportedly, ~96% of EPP patients is a rare pathogenic FECH variant in trans of a common intronic FECH variant c.315–48T>C. The homozygous mutations caused the insertion of 63 intronic base pairs and the nonsense-mediated decay of this aberrant mRNA molecule, thereby leading to sharply decreased FECH activity (15). The deficiency of the FECH enzyme results in the accumulation of predominantly protoporphyrin in the circulating erythrocytes and plasma (16). The accumulation of protoporphyrin causes the occurrence of EPP (17). The protoporphyrin molecules are photodynamic and photoactivated by the sun and trigger the activation of an inflammatory response due to the photodamage, including the complement system and the inflammatory factor, which leads to the damage of tissue and vessel. Thus, the hallmark of EPP is a painful photosensitivity reaction after exposure to sunlight. The pain is usually manifested as tingling, itching, and burning. Erythema, edema, or vesicle are commonly observed (18). Moreover, the photoactivation of the protoporphyrin molecules presumably lead to the release by formation of singlet oxygen and other oxygen radicals which further cause the expression of inflammatory mediators. Furthermore, the augmented presence of reactive oxygen species can contribute to further accumulation of DNA damage, thereby exacerbating skin sensitivity to ultraviolet radiation in individuals with XPA mutation and consequently intensifying the severity of cutaneous manifestations in XPA patients.

Studies (17) have pointed out that the diagnostic criteria for EPP are as follows: first, photosensitivity is the clinical basis for the diagnosis of EPP, which is characterized by skin pain and burning sensation after sun exposure; second, porphyrins and their derivatives can emit red fluorescence after absorption, and the plasma fluorescence peak value of EPP patients is close to 634 nm wavelength; and third, erythrocyte protoporphyrin increases, mainly without metal protoporphyrin, and urinary porphyrin is normal. In this reported case, urinary porphyrin was normal, and pemphigus and pemphigoid antibodies were negative. However, the total erythrocyte protoporphyrin and metal-free protoporphyrin IX were not further confirmed at the patient’s first visit. However, the histological examination showed significant vacuolar degeneration of basal cells and intercellular edema, which could be considered as supplementary evidence for the diagnosis of EPP.

Herein, we reported a young Chinese patient with XPA and EEP. XPA and EPP are rare photodermatoses, resulting in acute, painful phototoxicity on sun exposure. The presentation of cutaneous manifestations in XPA and EPP are similar, especially in the early stages of the disorders. Besides the typical cutaneous symptoms, the XPA patients often present with ophthalmological symptoms and idiopathic late-onset neurological syndromes. Moreover, medications for the treatment of XPA and EEP have shown limited or no efficacy. Various treatment strategies such as beta-carotene, cysteine, and vitamin C have been tried for EPP, although the evidence is limited. Afamelanotide, an analogue of alpha-melanocyte stimulating hormone, has been granted approval by the European Medicines Agency and the Food and Drug Administration for the treatment of EPP. It induces skin pigmentation and enhances tolerance to sunlight, while leaving unaffected the underlying metabolic defect or reducing levels of protoporphyrin (19). As for the treatment of XPA, no therapies can halt the progression of the disease and, therefore, management remains symptomatic. However, anti-inflammatory drugs reduce UV-induced inflammation and skin tumor development in XPA by suppressing the inflammatory response and oxidative stress after UV irradiation (20). Given the limited efficacy, effective management assumes paramount importance in individuals with XPA and EPP. Patients and their families should receive counseling on sun protection measures, including the utilization of protective clothing. It is recommended to schedule annual follow-up appointments that encompass laboratory investigations.

## Conclusion

XPA and EPP, rare photodermatoses, manifest erythema and edema in early childhood after short sun exposure. The presentation of cutaneous manifestations, similar in XPA and EPP, are patients’ main reason for consultation. However, the manifestations can sometimes be subtle and confusing, leading to significant diagnostic delays. Both XPA and EPP have profound effects on a patient’s family and social relationships and management such as avoidance of sun exposure seems to be the primary and vital mode of symptom prevention.

## Data availability statement

The original contributions presented in the study are included in the article/supplementary material. Further inquiries can be directed to the corresponding author.

## Ethics statement

The studies involving humans were approved by Ethics Committee of the Second Affiliated Hospital of Hunan University of Chinese Medicine. The studies were conducted in accordance with the local legislation and institutional requirements. Written informed consent for participation in this study was provided by the participants’ legal guardians/next of kin. Written informed consent was obtained from the individual(s), and minor(s)’ legal guardian/next of kin, for the publication of any potentially identifiable images or data included in this article.

## Author contributions

S-hW: Visualization, Writing – original draft. TX: Resources, Writing – review & editing. DZ: Data curation, Visualization, Writing – review & editing. Y-hZ: Resources, Writing – review & editing. M-fZ: Conceptualization, Resources, Writing – review & editing, Funding acquisition.

## References

[1] Kleijer WJ Laugel V Berneburg M Nardo T Fawcett H Gratchev A . Incidence of DNA repair deficiency disorders in western Europe: Xeroderma pigmentosum, Cockayne syndrome and trichothiodystrophy. DNA Repair (Amst). (2008) 7:744–50. doi: 10.1016/j.dnarep.2008.01.014 18329345

[2] Imani MM Basamtabar M Akbari S Sadeghi E Sadeghi M Relationship between XPA . XPB/ERCC3, XPF/ERCC4, and XPG/ERCC5 polymorphisms and the susceptibility to head and neck carcinoma: A systematic review, meta-analysis, and trial sequential analysis. Medicina (Kaunas). (2024) 60(3):478. doi: 10.3390/medicina60030478 38541204 PMC10972270

[3] Garcia-Moreno H Langbehn DR Abiona A Garrood I Fleszar Z Manes MA . Neurological disease in xeroderma pigmentosum: prospective cohort study of its features and progression. Brain. (2023) 146:5044–59. doi: 10.1093/brain/awad266 PMC10690019 38040034

[4] Lucero R Horowitz D . Xeroderma pigmentosum. Treasure Island (FL: StatPearls (2023). 31855390

[5] Zebian A Shaito A Mazurier F Rezvani HR Zibara K . XPC beyond nucleotide excision repair and skin cancers. Mutat Res Rev Mutat Res. (2019) 782:108286. doi: 10.1016/j.mrrev.2019.108286 31843141

[6] Brambullo T Colonna MR Vindigni V Piaserico S Masciopinto G Galeano M . Xeroderma pigmentosum: A genetic condition skin cancer correlated-A systematic review. BioMed Res Int. (2022) 2022:8549532. doi: 10.1155/2022/8549532 35898688 PMC9313971

[7] Poli A Frieri C Lefebvre T Delforge J Mirmiran A Talbi N . Management of erythropoietic protoporphyria with cholestatic liver disease: A case report. Mol Genet Metab Rep. (2023) 37:101018. doi: 10.1016/j.ymgmr.2023.101018 38053924 PMC10694760

[8] Ahmed jan NMasood S . Erythropoietic protoporphyria. Treasure Island (FL: StatPearls (2023). 33085288

[9] Zhou EY Wang H Lin Z Xu G Ma Z Zhao J . Clinical and molecular epidemiological study of xeroderma pigmentosum in China: A case series of 19 patients. J Dermatol. (2017) 44:71–5. doi: 10.1111/1346-8138.13576 27607234

[10] Kusakabe M Onishi Y Tada H Kurihara F Kusao K Furukawa M . Mechanism and regulation of DNA damage recognition in nucleotide excision repair. Genes Environ. (2019) 41:2. doi: 10.1186/s41021-019-0119-6 30700997 PMC6346561

[11] Quezada-Maldonado EM Chirino YI Gonsebatt ME Morales-Bárcenas R Sánchez-Pérez Y García-Cuellar CM . Nucleotide excision repair pathway activity is inhibited by airborne particulate matter (PM(10)) through XPA deregulation in lung epithelial cells. Int J Mol Sci. (2022) 23(4):2224. doi: 10.3390/ijms23042224 35216341 PMC8878008

[12] van den Heuvel D Kim M Wondergem AP van der Meer PJ Witkamp M Lambregtse F . A disease-associated XPA allele interferes with TFIIH binding and primarily affects transcription-coupled nucleotide excision repair. Proc Natl Acad Sci U S A. (2023) 120:e2208860120. doi: 10.1073/pnas.2208860120 36893274 PMC10089173

[13] Khalat N Messaoud O Ben Rekaya M Chargui M Zghal M Zendah B . First genetic characterization of Xeroderma pigmentosum in Libya: High frequency of XP-C founder mutation. Mol Genet Genomic Med. (2023) 11:e2158. doi: 10.1002/mgg3.2158 36812379 PMC10265042

[14] Brancaleoni V Granata F Missineo P Fustinoni S Graziadei G Di Pierro E . Digital PCR (dPCR) analysis reveals that the homozygous c.315-48T>C variant in the FECH gene might cause erythropoietic protoporphyria (EPP). Mol Genet Metab. (2018) 124:287–96. doi: 10.1016/j.ymgme.2018.06.005 29941360

[15] Oustric V Manceau H Ducamp S Soaid R Karim Z Schmitt C . Antisense oligonucleotide-based therapy in human erythropoietic protoporphyria. Am J Hum Genet. (2014) 94:611–7. doi: 10.1016/j.ajhg.2014.02.010 PMC3980518 24680888

[16] Chiara M Primon I Tarantini L Agnelli L Brancaleoni V Granata F . Targeted resequencing of FECH locus reveals that a novel deep intronic pathogenic variant and eQTLs may cause erythropoietic protoporphyria (EPP) through a methylation-dependent mechanism. Genet Med. (2020) 22:35–43. doi: 10.1038/s41436-019-0584-0 31273344

[17] Di Pierro E Granata F De Canio M Rossi M Ricci A Marcacci M . Recognized and emerging features of erythropoietic and X-linked protoporphyria. Diagnostics (Basel). (2022) 12(1):151. doi: 10.3390/diagnostics12010151 35054318 PMC8775248

[18] Dickey AK Leaf RK Balwani M . Update on the porphyrias. Annu Rev Med. (2024) 75:321–35. doi: 10.1146/annurev-med-042921-123602 37540847

[19] Balwani M Naik H Overbey JR Bonkovsky HL Bissell DM Wang B . A pilot study of oral iron therapy in erythropoietic protoporphyria and X-linked protoporphyria. Mol Genet Metab Rep. (2022) 33:100939. doi: 10.1016/j.ymgmr.2022.100939 36406817 PMC9672425

[20] Tsujimoto M Kakei Y Yamano N Fujita T Ueda T Ono R . Clinical trial on the efficacy and safety of NPC-15 for patients with xeroderma pigmentosum exaggerated sunburn reaction type: XP-1 study protocol for a multicentre, double-blinded, placebo-controlled, two-group crossover study followed by a long-term open study in Japan. BMJ Open. (2023) 13:e068112. doi: 10.1136/bmjopen-2022-068112 PMC10040004 36948554

